# Design of a reversible single precision floating point subtractor

**DOI:** 10.1186/2193-1801-3-11

**Published:** 2014-01-04

**Authors:** AV Anantha Lakshmi, GF Sudha

**Affiliations:** Department of Electronics and Communication Engineering, Pondicherry Engineering College, Puducherry, India

**Keywords:** Reversible comparator, Reversible floating point subtractor, Reversible leading zero detector, Reversible shift register, Reversible logic, FPGA

## Abstract

In recent years, Reversible logic has emerged as a major area of research due to its ability to reduce the power dissipation which is the main requirement in the low power digital circuit design. It has wide applications like low power CMOS design, Nano-technology, Digital signal processing, Communication, DNA computing and Optical computing. Floating-point operations are needed very frequently in nearly all computing disciplines, and studies have shown floating-point addition/subtraction to be the most used floating-point operation. However, few designs exist on efficient reversible BCD subtractors but no work on reversible floating point subtractor. In this paper, it is proposed to present an efficient reversible single precision floating-point subtractor. The proposed design requires reversible designs of an 8-bit and a 24-bit comparator unit, an 8-bit and a 24-bit subtractor, and a normalization unit. For normalization, a 24-bit Reversible Leading Zero Detector and a 24-bit reversible shift register is implemented to shift the mantissas. To realize a reversible 1-bit comparator, in this paper, two new 3x3 reversible gates are proposed The proposed reversible 1-bit comparator is better and optimized in terms of the number of reversible gates used, the number of transistor count and the number of garbage outputs. The proposed work is analysed in terms of number of reversible gates, garbage outputs, constant inputs and quantum costs. Using these modules, an efficient design of a reversible single precision floating point subtractor is proposed. Proposed circuits have been simulated using Modelsim and synthesized using Xilinx Virtex5vlx30tff665-3. The total on-chip power consumed by the proposed 32-bit reversible floating point subtractor is 0.410 W.

## Introduction

Power dissipation is an important factor in VLSI design. There are some classical approaches to reduce the dynamic power such as reducing supply voltage, decreasing physical capacitance and reducing switching activity. These techniques are not fit enough to meet today’s power requirement. Thus, most research has focused on building adiabatic logic, which is a promising design for low power applications. Adiabatic logic works with the concept of switching activities which reduces the power by giving stored energy back to the supply. Thus, the term adiabatic logic is used in low-power VLSI circuits which implements reversible logic. Conventionally digital circuits have been implemented using the basic logic gates which were irreversible in nature. These irreversible gates produce energy loss due to the information bits lost during the operation. Information loss occurs because total number of output signals generated is less than total number of input signals applied. Thus, conventional combinational logic circuits dissipate heat for every bit of information that is lost during their operation. Landauer (Landauer 1961) proved that a single bit of information loss dissipates KTln2 joules of energy where K is the Boltzmann’s constant and T is the temperature at which the computation is performed. Benett (Benett 1973) showed that in order to avoid energy loss it is necessary that all the computations have to be performed in a reversible way. Thus to avoid power dissipation, circuits must be constructed from reversible logic gates. Thus every future technology has to use reversible gates in order to reduce power dissipation. A circuit is said to be reversible if the input vector can be uniquely recovered from the output vector and if there is a one – to –one correspondence between its input and output assignment. A reversible circuit maps each input vector, into a unique output vector and vice versa. Reversible logic has application in various research areas such as digital signal processing, quantum computing, low power CMOS design, communication, bioinformatics and nanotechnology-based systems (Peres 1985). Synthesis of reversible logic circuits is significantly more complicated than traditional irreversible logic circuits because in a reversible logic circuit, we are not allowed to use fan-out and feedback (Perkowski et al. 2001). A reversible logic circuit should have the following features (Perkowski and Kerntopf 2001):

- Use minimum number of reversible gates
- Use minimum number of garbage outputs
- Use minimum constant inputs

The output which cannot be used further for computation process is known as garbage output. The input that is added to an nxk function to make it reversible is called constant input (Thapliyal and Srinivas 2005). The quantum cost of a reversible or quantum circuit is defined as the number of 1 × 1 or 2 × 2 gates used to implement the circuit. The major objective of a reversible logic design is to minimize the quantum cost and the number of garbage outputs (Benett 1998). Hence, one of the major issues in reversible circuit design is garbage minimization to minimise the power dissipation. Another significant criterion in designing a reversible logic circuit is to minimize the number of reversible gates used (Haghparast and Navi 2008). In this paper, it is proposed to design an efficient reversible single precision floating point subtractor. The proposed floating point subtractor design requires an efficient reversible comparator unit, an 8-bit and a 24-bit reversible subtractor unit, a 24-bit reversible leading zero detector unit and a 24-bit reversible shift register for normalization. The paper also focuses on the design of a reversible 1-bit comparator using the two newly proposed reversible gates Reversible Gate1 (RG1) and Reversible Gate2 (RG2). Since in adiabatic circuits energy is reused rather than just dissipated, the transistor representation of the proposed reversible gates RG1 and RG2 are implemented using adiabatic logic. Also, an eight-bit and a 24 –bit reversible comparator is designed using the 1-bit comparator. An 8-bit and a 24-bit reversible subtractor is implemented using the TR gate. Normalization unit requires an efficient reversible leading zero detector and a 24-bit reversible shift register. A reversible leading zero detector unit is designed using the Reversible Gate1 (RG1)and a 24-bit reversible shift register is implemented using the Fredkin, Feynman and NOT gate. All the proposed circuits have been implemented using VHDL and simulated using Modelsim. The paper is organized as follows: Reversible Logic gates section overviews some of the reversible gates used in the literature. Floating Point Subtraction Algorithm section briefly explains the steps involved in floating point subtraction. Related work section overviews some of the recent relevant methods in the literature. Proposed Reversible gates section introduces the two new reversible gates (RG1 and RG2). Transistor representation of the Proposed Reversible gates using Adiabatic Method section briefly explains the transistor schematic of the proposed gates (RG1 and RG2) using adiabatic logic. Proposed design of Reversible single precision floating point subtractor section explains the efficient realization of 1-bit, an 8-bit and a 24-bit comparator using the two proposed gates RG1 and RG2, realization of an 8-bit and a 24-bit subtractor using TR gates, realization of a 24-bit reversible leading zero detector unit using the proposed gate RG1 and the design of 24-bit reversible shift register. Results obtained from the proposed gates are presented in simulation results section. Device utilization summary is presented in synthesis reports section. Finally, Conclusion section summarizes the main conclusions of this work and indicates directions for future work.

### Reversible logic gates

Some of the important reversible logic gates are Feynman gate, Fredkin gate, Toffoli gate, Peres gate, URG gate, BJN gate, TR gate, M gate and L gate. Brief introduction of these gates are as shown in Table 1.

**Table 1:** **Reversible logic gates**

SI.No	Gate	Block diagram	Function
1.	Feynman		P = A
			Q = *A* ⊕ *B*
2.	Toffoli		P = A
			Q = B
			R = *AB* ⊕ *C*
3.	URG		P = (*A* + *B*) ⊕ *C*
			Q = B
			R = *AB* ⊕ *C*
4.	TR		P = A
			Q = *A* ⊕ *B*
			R = *AB*′ ⊕ *C*
5.	BJN		P = A
			Q = B
			R = (*A* + *B*) ⊕ *C*
6.	Fredkin		P = A
			Q = A’B + AC
			R = AB + A’C
7.	Peres		P = A
			Q = *A* ⊕ *B*
			R = *AB* ⊕ *C*
8.	M		P = A
			Q = (*A* ⊕ *B*)′
			R = *AB*′ ⊕ *C*
9.	L		P = A
			Q = B
			R = (*A* + *B*)′ ⊕ *C*

### Floating point subtraction algorithm overview

**Figure 1:**
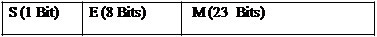
**IEEE 754 standard of a single precision floating point number.**

Floating point numbers are one possible way of representing real numbers in binary format; the IEEE 754. This work focuses only on single precision floating point format. Figure 1 shows the IEEE 754 standard of a single precision floating point number.

The single precision floating point number has three fields: a single sign bit S, an eight bit biased exponent E, and a twenty three bit fraction Mantissa M. The floating point subtraction of two numbers can be expressed as,1

The operations involved in floating point subtraction are (a) aligning the binary point, (b) subtraction of aligned mantissas and (c) normalization of the result. The detailed steps are as follows:

1. Compare the mantissa of the operands.
2. Align binary point:
  Initial result exponent: the larger of A_e_, B_e_
  Compute exponent difference: B_e_ - A_e_
  If B_e_ > = A_e_ Right shift A_m_ that many positions to form A_m_ 2 ^Ae-Be^
  If A_e_ > B_e_ Right shift B_m_ that many positions to form B_m_ 2 ^Be-Ae^
3. Subtract the aligned mantissas: i.e. A_m_2 ^Ae-Be^ - B_m_ or A_m_ - B_m_2 ^Be-Ae^
4. If normalization of result is needed, then a normalization step follows:
  If ((A_e_ ! = B_e_ and bout = 0) or if A_e_ = B_e_), then left shift result, decrement result exponent (e.g., if result is 0.001xx…)
  Else if A_e_ ! = B_e_ and bout = 1, then Right shift result, increment result exponent (e.g., if result is 10.1xx…) Continue until MSB of data is 1
5. Check result exponent:
  If larger than maximum exponent allowed return exponent overflow.
  If smaller than minimum exponent allowed return exponent underflow.
  From the steps involved in floating point subtraction, the following observations are made:
    i. To align the binary point, the initial exponent result should be larger of A_e_, B_e_. To realize this, an 8- bit reversible comparator is required to compare the two exponents.
    ii. To determine A_e_ – B_e_ or B_e_ – A_e_, an eight bit reversible subtractor is required to be designed.
    iii. To align the mantissas, A_m_ or B_m_ needs to be shifted for which a 24-bit reversible shift register or a 25-bit reversible shift register is needed to include the guard bits.
    iv. To subtract the mantissas, a 24-bit reversible subtractor is to be designed.
    v. To determine the sign part, a 24-bit reversible comparator to compare the mantissa is required.
    vi. For normalization of the result, a 24-bit reversible leading zero detector is to be designed.

Thus the floating point subtraction unit requires the implementation of an 8-bit reversible comparator, a 8-bit reversible subtractor, a 24-bit reversible shift register, a 25-bit reversible shift register, a 24-bit reversible subtractor, a 24-bit reversible comparator and a 24-bit reversible leading zero detector.

### Related work

A reversible one-bit comparator is realized using the existing reversible gates such as Fredkin, Peres, Toffoli, R, URG, TR and the newly proposed gate BJN (Nagamani et al. 2011). The drawback of their work is that the number of reversible gates required for each implementation is more. Also it produces more number of garbage outputs and the circuit uses more number of constant inputs. Another work on reversible one – bit comparator is designed using a single SCG gate (Digantha et al. 2011). The number of garbage outputs produced is 1. It uses 2 constant inputs. The transistor representation of their circuit is not given. Since the logical expressions involved in SCG is complex, definitely it requires more number of transistors to implement. To minimize the transistor count, we have proposed two new 3×3 reversible gates which can be combined for its use as a reversible 1-bit comparator. Few works were reported on reversible half subtractors. A reversible half subtractor is realized by Murali (Murali et al. 2002). The drawback of this work is that the critical path delay of a single reversible gate is 4. A work on reversible binary subtractors is carried out using new reversible TR gate (Thapliyal 2011). The number of reversible gates required is one and the critical path delay associated with this circuit is 4. Hence, we have designed a reversible full subtractor using two TR gates. Only few works were reported on reversible sequential elements. A work on reversible D – flip-flop contains four New gates and one Feynman gate, a total of 7 reversible gates and produces 8 garbage outputs (Sivakumar et al. 2006). Another work on reversible master–slave D-flip-flop is reported by Noor Muhammed Nayeem, Lafifa Jamal and Hafiz Md. Hasan Babu (Noor Muhammed et al. 2009). The number of reversible gates required is 5 and the number of garbage outputs produced by this circuit is 2. The circuit is minimized in terms of gate count and garbage outputs.

### Proposed reversible gates (RG1 and RG2)

#### Proposed Reversible Gate1 (RG1)

**Figure 2:**
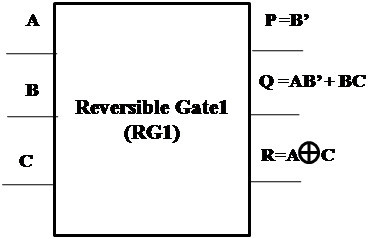
**Proposed 3x3 Reversible Gate1 (RG1).**

The logic diagram of the proposed new 3×3 Reversible Gate1 (RG1) is as shown in Figure 2. Reversible Gate1 is a 3×3 gate with inputs (A,B,C) and outputs P = B’, Q = AB’ + BC and R = *A* ⊕ *C*.

The truth table for the corresponding gate is as shown in Table 2.

**Table 2:** **Truth table of Reversible Gate 1 (RG1)**

A	B	C	P	Q	R
0	0	0	1	0	0
0	0	1	1	0	1
0	1	0	0	0	0
0	1	1	0	1	1
1	0	0	1	1	1
1	0	1	1	1	0
1	1	0	0	0	1
1	1	1	0	1	0

A closer look at the Truth Table reveals that the input pattern corresponding to a specific output pattern can be uniquely determined and thereby maintaining that there is a one-to-one correspondence between the input vector and the output vector. In this gate the input vector is given by IV = (A,B,C) and the corresponding output vector is OV = (P,Q,R). The quantum cost of the proposed Reversible Gate1 (RG1) is 5. The quantum cost is calculated based on the number of 1×1 and 2×2 primitive operations.

#### Proposed Reversible Gate2(RG2)

**Figure 3:**
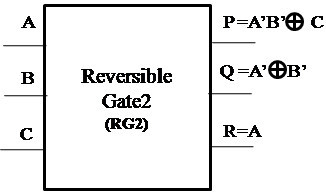
**Proposed 3x3 Reversible Gate2 (RG2).**

The logic diagram of the proposed new 3×3 Reversible Gate2 (RG2) is as shown in Figure 3. Reversible Gate2 is a 3×3 gate with inputs (A,B,C) and outputs P = *A*′*B*′ ⊕ *C*, *Q* = *A*′ ⊕ *B*′ and R = A.

The truth table for the corresponding gate is as shown in Table 3.

**Table 3:** **Truth table of Reversible Gate2 (RG2)**

A	B	C	P	Q	R
0	0	0	1	0	0
0	0	1	0	0	0
0	1	0	0	1	0
0	1	1	1	1	0
1	0	0	0	1	1
1	0	1	1	1	1
1	1	0	0	0	1
1	1	1	1	0	1

A closer look at the Truth Table reveals that the input pattern corresponding to a specific output pattern can be uniquely determined and thereby maintaining that there is a one-to-one correspondence between the input vector and the output vector. In this gate the input vector is given by IV = (A,B,C) and the corresponding output vector is OV = (P,Q,R). The quantum cost of the proposed Reversible Gate2 (RG2) is 5.

#### Realization of the classical operations

##### Proposed Reversible Gate1 (RG1)

**Figure 4:**
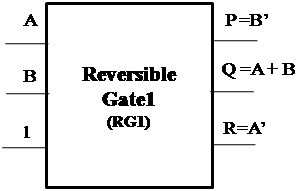
**Reversible Gate1 implementing reversible OR operation.**

**Figure 5:**
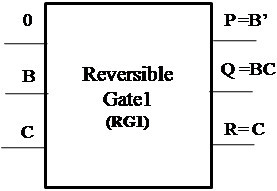
**Reversible Gate1 implementing reversible AND operation.**

**Figure 6:**
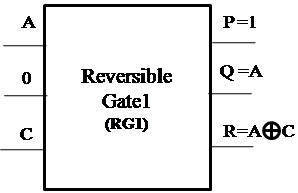
**Reversible Gate1 implementing reversible XOR operation.**

**Figure 7:**
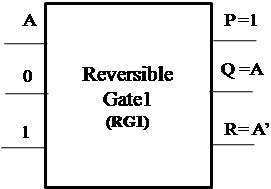
**Reversible Gate1 implementing reversible NOT and COPY operation.**

The proposed Reversible Gate1 (RG1) can implement OR, AND, XOR, NOT and COPY operation. Also the COPY operation is an important operation which can be realized using the proposed Reversible Gate1. The classical operations realized using Reversible Gate1 are shown in Figures 4, 5, 6 and 7.

#### Proposed Reversible Gate2 (RG2)

**Figure 8:**
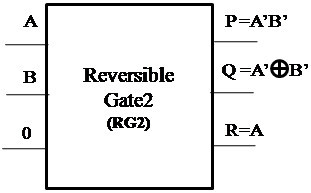
**Reversible Gate2 implementing reversible NOR operation.**

**Figure 9:**
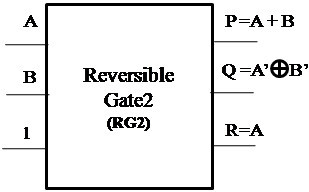
**Reversible Gate2 implementing reversible OR operation.**

**Figure 10:**
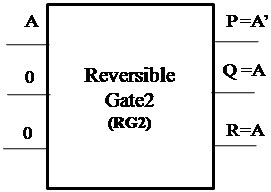
**Reversible Gate2 implementing reversible NOT and COPY operation.**

The proposed Reversible Gate2 can implement OR, NOR, NOT and COPY operation. Also the COPY operation is an important operation which can be realized using the proposed Reversible Gate2. The fact that the proposed gate can implement NOR operation signifies that any boolean function can be implemented using the gate as NOR gate is a universal gate. The classical operations realized using Reversible Gate2 are shown in Figures 8, 9 and 10.

### Transistor representation of the proposed reversible gates using adiabatic method

Adiabatic circuits are those circuits which recycles the energy from output nodes instead of discharging it to ground. In literature, adiabatic logic circuits classified into two types: full adiabatic and quasi or partial adiabatic circuits. Full-adiabatic circuits have no non-adiabatic loss, but they are much more complex than quasi-adiabatic circuits. Quasi-adiabatic circuits have simple architecture and power clock system. There are two types of energy loss in quasi-adiabatic circuits, adiabatic loss and non-adiabatic loss. The adiabatic loss occurs when current flows through non-ideal switch, which is proportional to the frequency of the power-clock. If any voltage difference between the two terminals of a switch exists when it is turned on, non-adiabatic loss occurs. The non-adiabatic loss, which is independent of the frequency of the power-clock, is proportional to the node capacitance and the square of the voltage difference. Several quasi-adiabatic logic architectures have been reported, such as ECRL, 2 N-2N-2P, PFAL etc. (Athas et al. 1994; Dickinson et al. 1995; Alioto et al. 2001; Fischer et al. 2004; Kime et al. 2005; Anuar et al. 2009; Yadav et al. 2011). In adiabatic systems, more than one power clock is required. We have proposed the transistor representation of the proposed gate Reversible Gate1 and Reversible Gate2 using ECRL technique. In the proposed work, three phases of power clock is used to achieve the synchronization and the input signal is phase shifted by 90^°^ with respect to the power clock.

### Transistor representation of proposed Reversible Gate1 (RG1)

**Figure 11:**
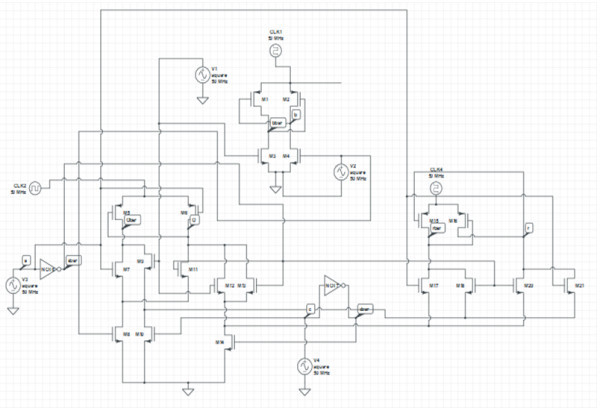
**Transistor representation of proposed Reversible Gate1 (RG1).**

The transistor representation of proposed Reversible Gate1using adiabatic logic is shown in Figure 11.

If the input B is 0, then the transistor M4 conducts and the output node b will be pulled to logic 0. When the node b is 0, the transistor M1 conducts and the output node bbar follows the clock pulse CLK1. When B is 1, then M3 conducts and the output node bbar will be pulled to logic 0. When the node bbar is 0, the transistor M2 conducts and the output node b follows the clock pulse CLK1. Thus, the transistors M1, M2, M3 and M4 represent the inverter i.e. the function P. The transistors M5, M6, M7, M8, M9, M10, M11, M12, M13 and M14 represent the function ab’ + bc i.e. the function Q. The transistors M15, M16, M17, M18, M20, M21 represent the function a XOR c i.e. the function R. Thus a total of 20 transistors are required to implement the function of Reversible Gate1 using adiabatic logic. The average power dissipated by the gate at a frequency of 50 MHz is 108 mW while the same implementation using CMOS GDI logic dissipates a power of 30 W at a frequency of 50 MHz.

### Transistor representation of proposed Reversible Gate2 (RG2)

**Figure 12:**
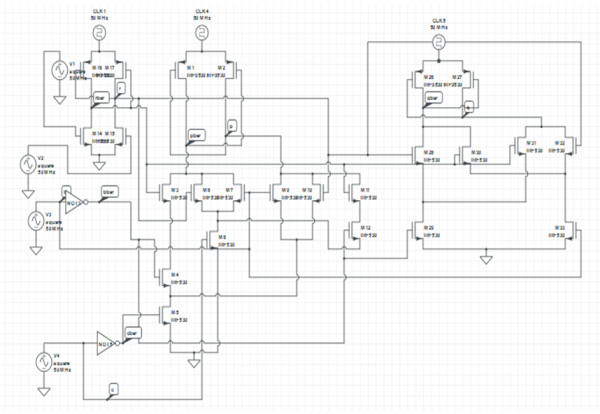
**Transistor representation of proposed Reversible Gate2 (RG2).**

The transistor representation of Proposed Reversible Gate2 using adiabatic logic is shown in Figure 12.

The transistors M1, M2, M3, M4, M5, M6, M7, M8, M9, M10, M11 and M12 represent the function (a’b’) XOR c i.e. the function P. The transistors M14, M15, M16 and M17 represent the function of inverter i.e. the function R. The transistors M26, M27, M28, M29, M30, M31, M32 and M33 represent the function a XOR b i.e. the function Q. Thus, a total of 24 transistors are required to implement the Reversible Gate2 using adiabatic logic. The average amount of power dissipated by the proposed implementation at a frequency of 50 MHz is 113 mW while the same implementation using CMOS GDI logic dissipates a power of 40 W at 50 MHz.

### Proposed design of reversible single precision floating point subtractor

#### Realization of one-bit comparator

To minimise the transistor count, a reversible one-bit comparator is implemented using the proposed reversible Gates1 and 2 (RG1 and RG2).

**Figure 13:**
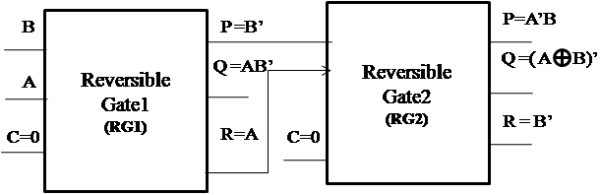
**Symbolic representation of proposed reversible one-bit comparator using RG1 and RG2.**

The symbolic representation of the proposed reversible one-bit comparator is shown in Figure 13.

From reversible Gate1 (RG1), when C is 0, P = B’, R = A and Q = AB’ which represents the greater function. The outputs P and R from reversible gate1 are given as the inputs A and B to the reversible gate2. From reversible gate2 (RG2), when C is 0, P = A’B which represents that A < B, Q = A XNOR B which represents that A = B and R = A which is the garbage output. Thus, the proposed one-bit comparator circuit requires 2 reversible gates. The circuit accepts 2 constant inputs and produces one garbage output which is an optimized circuit. The number of transistors required to implement the proposed circuit is 44.

#### Realization of 8-bit comparator

##### Logic diagram to find the greater of two 8-bit numbers

**Figure 14:**
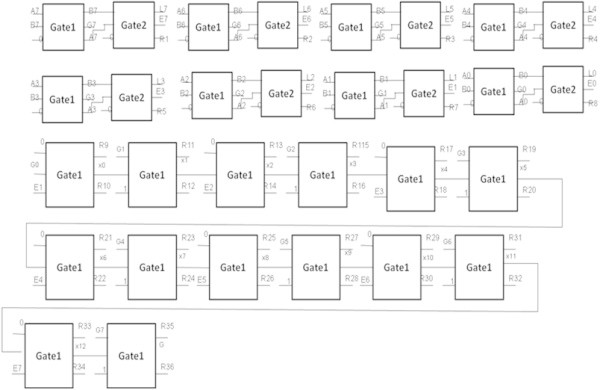
**Logic diagram for finding the greater of two eight-bit numbers.**

Let the two 8 – bit numbers to be compared for match be A = A7A6A5A4A3A2A1A0 and B = B7B6B5B4B3B2B1B0. Each pair of bits (AiBi) is fed to each comparator as shown in Figure 14. Consequently each comparator block compares two input bits and generates the respective outputs Ei, Gi and Li. The naming of the outputs is as follows:

Ei shows True logic when Ai = Bi,

Gi shows True logic when Ai > Bi,

Li shows True logic if Ai < Bi. For efficient realization of the greater of the two numbers, the Gi outputs are employed using the following logical Equation as proposed by Digantha (Digantha et.al 2011).

On simplification the above expression can be expressed as2

Close observation of (2) reveals that only fourteen reversible gate1 are sufficient to implement the logic, seven working as AND gates and the rest seven as OR gates. It has already been seen in Figures 4 and 5 that a reversible gate1 is capable of implementing the OR operation as well as the AND operation. Figure 14 shows the implementation of (2) for finding out the greater of the two eight-bit numbers A and B.

Figure 14 shows the logic diagram for finding the greater of two eight-bit numbers.

G0,G1,G2,G3,G4,G5,G6,G7,E0,E1,E2,E3,E4,E5,E6,E7,L0,L1,L2,L3,L4,L5,L6 and L7 are the outputs of the one-bit comparator block. The signal G = G7 + E7 (G6 + E6 (G5 + E5 (G4 + E4 (G3 + E3 (G2 + E2 (G1 + E1G0)))))) will be high when the input A is greater than B which is represented by signal G and the signals R1, R2, ...., R36 represent the garbage output. Table 4 shows the number of gates required to find the greater of two eight-bit numbers.

**Table 4:** **Number of gates required to find the greater of two eight-bit numbers**

SI. No	Number of gates required	Number of garbage outputs produced
1. Proposed work	30	36

### Logic diagram to find the equality of two 8-bit numbers

**Figure 15:**
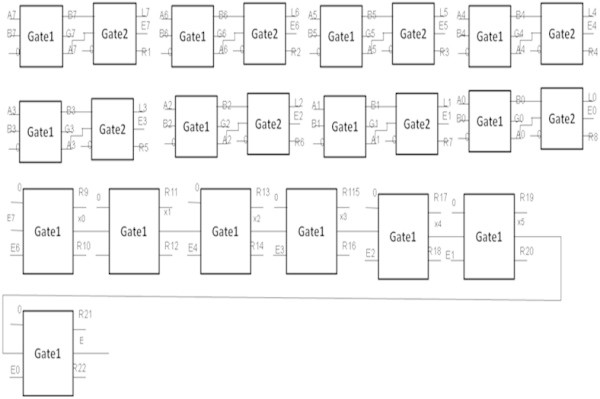
**Logic diagram for finding the equality of two eight-bit numbers.**

Figure 15 shows the logic diagram for finding the equality of two eight-bit numbers.

G0,G1,G2,G3,G4,G5,G6,G7,E0,E1,E2,E3,E4,E5,E6,E7,L0,L1,L2,L3,L4,L5,L6 and L7 are the outputs of the one-bit comparator block. The signal E = E7E6E5E4E3E2E1E0 will be high when the input A is equal to B. R1, R2, ...., R22 represents the garbage output. Table 5 shows the number of gates required to implement the equality of two eight-bit numbers.

**Table 5:** **Number of gates required to find the equality of two eight-bit numbers**

SI. No	Number of gates required	Number of garbage outputs produced
1. Proposed work	23	22

### Logic diagram to find the smaller of two 8-bit numbers

Since it can be detected whether two 8-bit numbers are equal or a number is greater than the other, it can be inferred that the architecture of whether a number is smaller than the other can also be designed simply by the logical expression:3

**Figure 16:**
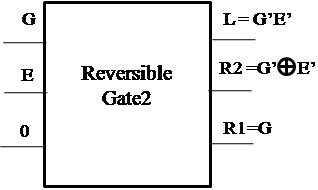
**Logic diagram for finding the smaller of two eight-bit numbers.**

Thus two outputs of Figures 14 and 15 are fed as the inputs to a reversible gate2 as shown in Figure 16.

In Figure 16, the signal L will be high when the input A is smaller than B. R1 and R2 represents the garbage output.

Table 6 shows the number of gates required to implement the reversible eight-bit comparator circuit (greater, smaller and equal).

**Table 6:** **Number of gates required to implement an eight-bit reversible comparator**

SI. No	Number of gates required	Number of garbage outputs produced
1. Proposed work	26	36

### Realization of 24-bit comparator

The two 24-bit operands A and B are fed as inputs to 24 one-bit comparators. Thus, a reversible 24-bit comparator is designed by using twenty four one-bit comparators. The twenty four 1-bit comparators are used to generate the greater (G0 – G23), equal (E0-E23) and the smaller signals (L0-L23) of the given two 24-bit numbers.

### Logical condition for the two 24-bit numbers to be equal

The two 24-bit numbers will be equal if the following condition is satisfied.4

To realize this condition, twenty three Reversible Gate1s (RG1) are used to generate the AND term.

### Logical condition for finding the greater of two 24-bit numbers

For efficient realization of the greater of the two numbers, the G output is employed using the following logical Equation.5

To realize Equation 5, twenty three reversible AND gates and twenty three reversible OR gates are required. Reversible AND and OR function is realized using Gate1(RG1)

### Logical condition to find the smaller of the two 24-bit numbers

The smaller of the given two 24-bit numbers can be realized by the following logical condition.6

Thus the two outputs E and G are fed as the inputs to a reversible gate2.

Table 7 shows the total number of gates required to implement the reversible 24-bit comparator circuit (greater, smaller and equal) using our two proposed gates RG1 and RG2.

**Table 7:** **Number of gates required to implement a 24-bit reversible comparator**

SI. No	Number of gates required	Number of garbage outputs produced
1. Proposed work	94	139

### Realization of 8-bit subtractor

**Figure 17:**
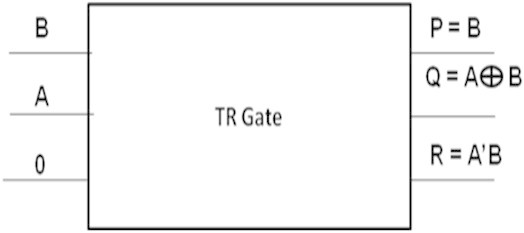
**Symbolic representation of TR Gate as a half subtractor.**

Realization of an efficient half subtractor using TR gates with critical path delay of 4 has been proposed in (Thapliyal and Ranganathan 2011). In this work, it is proposed to design a reversible eight-bit subtractor using the reversible full subtractors and the reversible half subtactor with TR gates. The symbolic representation of a reversible half subtractor using TR gates is shown in Figure 17.

**Figure 18:**
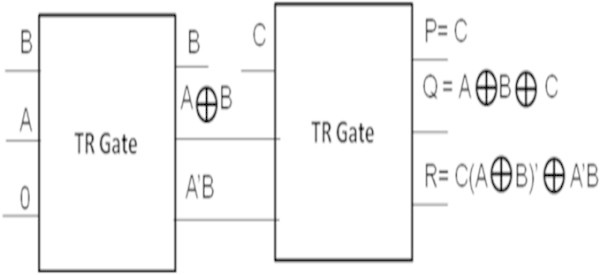
**Symbolic representation of TR Gate as a full subtractor.**

The symbolic representation of a reversible full subtractor using two TR gates is shown in Figure 18.

In this work, the reversible eight-bit subtractor is designed by cascading one reversible half subtractor and seven reversible full subtractors. Table 8 shows the number of gates required to implement an eight-bit reversible subtractor.

**Table 8:** **Number of gates required to implement an eight-bit reversible subtractor**

SI. No	Number of gates required	Number of garbage outputs produced
1. Proposed work	15	15

Thus, the number of gates required to implement an 8-bit reversible subtractor is 15.

### Realization of reversible 24-bit subtractor

Similarly, the reversible 24-bit subtractor is designed by cascading a reversible half subtractor and 23 reversible full subtractors.

Table 9 shows the number of gates required to implement a 24-bit reversible subtractor.

**Table 9:** **Number of gates required to implement a 24-bit reversible subtractor**

SI. No	Number of gates required	Number of garbage outputs produced
1. Proposed work	47	47

Thus, the number of gates required to implement a 24-bit reversible subtractor is 47.

### Proposed design of reversible 24-bit leading zero detector

The general procedure for normalization unit of floating point arithmetic is as follows:

- Determination of leading zeros
- Shifting the resultant fraction (left/right)
- Incrementing/Decrementing the exponent based on the shift-operation
- Exception Handling

Hence, a reversible leading zero detector unit is designed using the proposed reversible gate RG1 which detects the number of leading zeros on the mantissa part and then mantissa is shifted left-wise that many number of times. To do so, 23 reversible gates RG1 are cascaded to perform an AND function which determines the all zero case. Based on the AND function output, leading zeros are determined. The output of the arithmetic unit is then shifted to normalize the result (Number of shifts is determined by the zero leading detector), followed by rounding. Thus, the leading zero output is then passed on to the exponent adjustment unit for normalization.

### Realization of reversible exponent adjustment unit for normalization

After the subtraction, the result may have a number of leading zero bits or have one more bit with value of one at the most significant bit (MSB). The normalization is needed to adjust the result so that it conforms to the floating-point number format. In normalization, if a shift is required, it is either a one place right shift or a multiple-place left shift. If the MSB has a value of one, one place of right shift takes place and the 8-bit exponent is passed through a reversible conditional increment unit. To do so, a 25-bit reversible right shift register is designed to include the guard bit to yield the correct result. Otherwise, one or several places of left shift is needed in conjunction with a corresponding decrement of the 8-bit exponent. Thus, a reversible 8-bit Subtractor unit using TR gates is used to adjust the output exponent depending on the number of shifts required. For that, a 24-bit reversible left-shift register is designed using the existing reversible D-flip-flop to be discussed in the next section.

### Realization of 24-bit shift register

**Figure 19:**
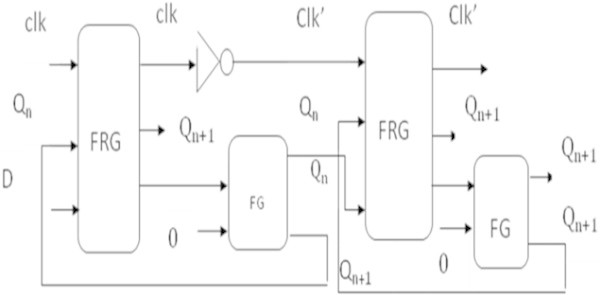
**Reversible master–slave D-flip-flop.**

The shift operation is performed using the sequential logic. The first step in the design of a reversible shift register is to design a reversible D-flip-flop. To design a 24-bit reversible shift register, 24 master–slave D-flip-flops are used as proposed by (Noor Muhammed et al. 2009). Figure 19 shows the implementation of a master–slave D-Flip-Flop using the reversible gates.

Each clock pulse shifts the contents of the register one bit position to the left. Each reversible master–slave D-flip-flop contains two Fredkin gates, two Feynman gates and one reversible NOT gate, a total of five gates and produces three garbage outputs. In reversible shift register, clk’ output of a D-flip-flop is connected to a reversible NOT gate and the inverted output is connected to the clk input of the next D-flip-flop. Therefore, reversible shift register reduces one garbage output from each D-flip-flop except the last one. Table 10 shows the comparison of the 24-bit reversible shift register.

**Table 10:** **Comparison of the 24 –bit reversible shift register**

	No. of gates	Garbage output
H. Thapliyal and M. Zwolinski (2006)	161	184
Proposed design	120	49
Improvement	26%	73%

It is seen that the proposed design has better performance compared to the existing work. The garbage outputs are 184 in the case of the existing work and 49 in the case of the proposed design i.e., an improvement of 73% in the proposed design compared to the existing work. The number of reversible gates required in the proposed design is 120 while in the case of the existing work is 161i.e an improvement of 26%.

### Realization of 25-bit right shift register

Similarly, to design a 25-bit reversible shift register, 25 master–slave D-flip-flops are used. Table 11 shows the number of gates required to implement a 25-bit Reversible shift register.

**Table 11:** **Number of gates required to implement a 25 –bit reversible shift register**

SI. No	Number of gates required	Number of garbage outputs produced
1. Proposed work	125	51

Table 12 summarizes the number of gates required, constant inputs required, garbage outputs produced and quantum cost to implement the proposed reversible single precision floating point subtractor.

**Table 12:** **Number of gates required for each module of proposed reversible single precision floating point subtractor**

	Number of gates required	Number of constant inputs	Number of garbage outputs produced	Quantum cost
24-bit subtractor	47	24	47	142
8-bit subtractor	15	8	15	46
24-bit comparator	118	94	163	590
8-bit comparator	34	26	44	170
24- bit left shift register	120	48	49	312
25- bit right shift register	125	50	51	325
24-bit leading zero detector	23	23	46	115
Exponent adjustment unit for normalization (includes 24 8-bit subtractors and 24 1-bit left shift registers)	475	240	407	1392
Total	957	513	822	3092

### Simulation results and discussion

**Figure 20:**
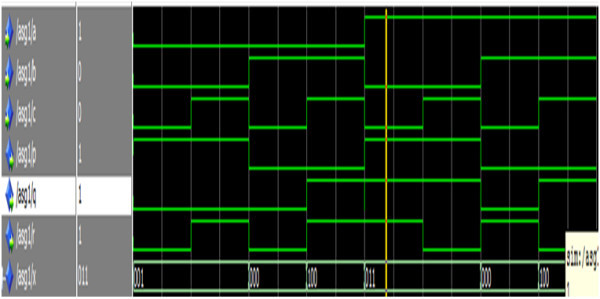
**Simulation result of the reversible Gate1.**

The entire unit was functionally verified. A test-bench is used to generate the stimulus and applies it to the implemented reversible 1-bit, 8-bit, 24- bit comparator, 8-bit, 24-bit reversible subtractor, 24-bit, 25-bit reversible shift register and 32-bit floating point subtractor. All the design units are functionally verified. The entire unit was implemented using structural style of modelling. The design was simulated using Modelsim and synthesized using Xilinx Virtex5 and the power is estimated using Xilinx Power Estimator tool. Figure 20 shows the simulation result of the reversible Gate1.

The signals a, b, c represents the input signals and p, q, r represents the output signals. From the truth table of reversible gate1 when the inputs a =1,b = 0 and c = 0, the corresponding outputs must be p = 1, q =1 and r = 1 which is depicted in Figure 20.

**Figure 21:**
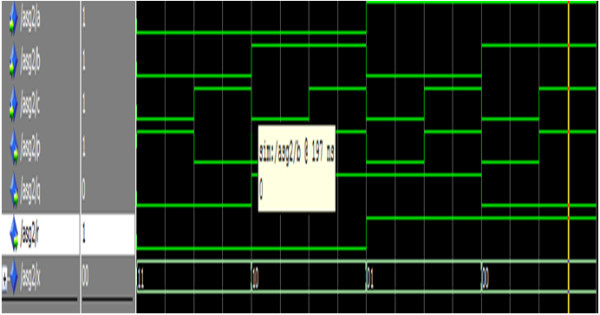
**Simulation result of the reversible Gate2.**

Figure 21 shows the simulation result of the reversible Gate2.

The signals a, b, c represents the input and p, q, r represents the output. From the truth table of reversible gate2 when the inputs a =1,b = 1 and c = 1, the corresponding outputs must be p = 1, q =0 and r = 1 which is depicted in Figure 21.

**Figure 22:**
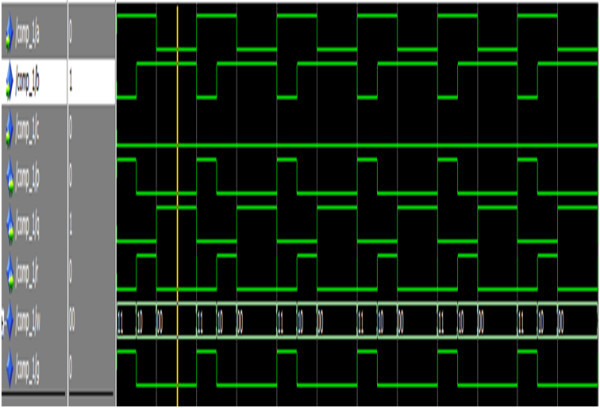
**Simulation result of the reversible one-bit comparator.**

Figure 22 shows the simulation result of the reversible one-bit comparator.

The signals a,b,c represents the input and p, q, r represents the output signals where p denotes the greater condition, q represents the smaller condition and r denotes the equality condition. Thus for the input combination a = 0, b = 1 and c = 0, the outputs are p = 0,q = 1 and r = 0. Thus the result indictes that a is smaller than b.

**Figure 23:**
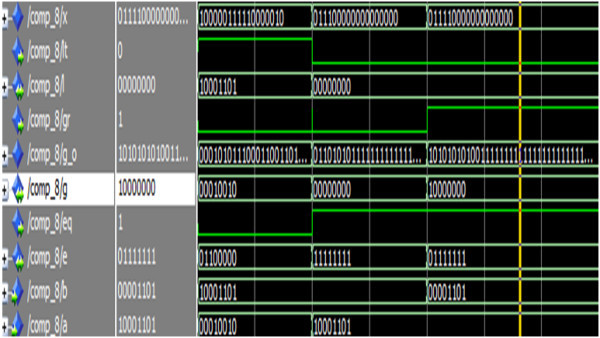
**Simulation result of the reversible 8-bit comparator.**

Figure 23 shows the simulation result of the reversible eight-bit comparator.

The operands a,b represents the two eight-bit numbers and the signals gr,eq and lt represents the output signals to indicate the greater, equality and lesser condition. For the input a = 10001101, b = 10001101, the outputs are gr = 0, eq = 1 and lt = 0. Thus the simulation result indicates that a is equal to b.

**Figure 24:**
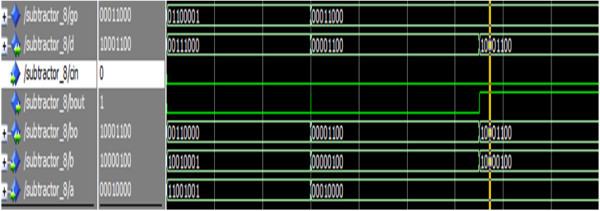
**Simulation result of the reversible 8-bit subtractor.**

Figure 24 shows the simulation result of the eight-bit reversible subtractor.

The operands a,b represents the two eight-bit numbers, cin is the primary carry/borrow input signal and the signals d, bo represents the eight-bit difference and borrow signals, bout is the primary borrow-out signal. For the input a = 00010000, b = 00000100, d = 00001100, bout = 0. Thus the simulation result depicts the implementation of eight-bit subtractor.

**Figure 25:**
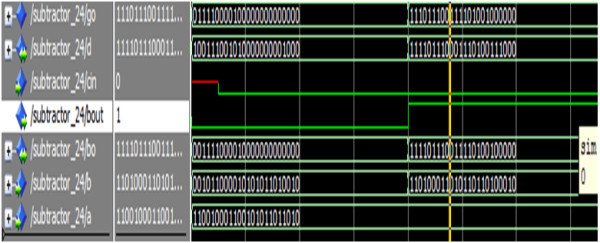
**Simulation result of the reversible 24-bit subtractor.**

Figure 25 shows the simulation result of the 24-bit reversible subtractor.

The operands a,b represents the two 24-bit numbers, cin is the primary carry/borrow input signal and the signals d, bo represents the 24-bit difference and borrow signals, bout is the primary borrow-out signal. For the input a = 110010001100101011011010 b = 001011000010101011010010, d = 100111001010000000001000, bout = 0. Thus the simulation result depicts the implementation of 24-bit subtractor.

**Figure 26:**
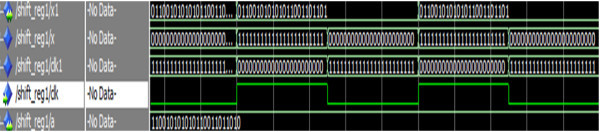
**Simulation result of the reversible 24-bit shift register.**

Figure 26 shows the simulation result of the 24-bit reversible shift register.

The operand a represents the 24-bit number to be shifted, clk is the primary clock signal and the signal ×1 represents the 24-bit shifted output. For the input a = 110010101010110011011010, ×1 = 011001010101011001101101 which is the shifted output of the signal a.

**Figure 27:**
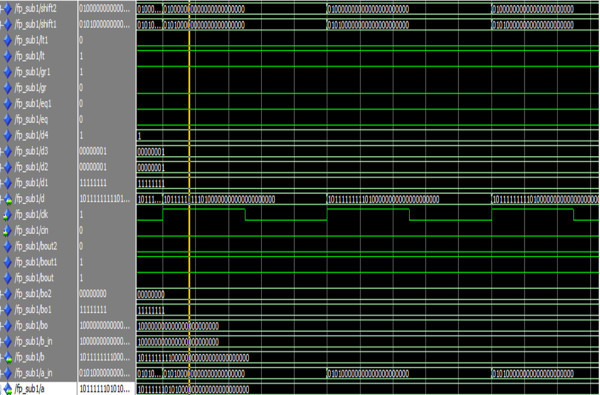
**Simulation result of the reversible single precision floating point subtractor.**

Figure 27 shows the simulation result of the reversible single precision floating point subtractor.

The operands a and b represents the two 32-bit floating point numbers. The signals gr,lt and eq represents the greater, smaller and equal conditions of a reversible eight-bit comparator. The signals gr1,lt1 and eq1 represents the greater, smaller and equal conditions of the reversible 24-bit comparator. The signals shift1 and shift2 represents the shifted versions of the mantissas a and b.The signal d represents the 32-bit floating point subtractor result.

### Synthesis report

The entire design has been synthesized using Xilinx Virtex5vlx30tff665-3. Each module is implemented using dataflow style of modelling. Then the top design is implemented using structural style of modelling.

Table 13 shows the device utilization summary.

**Table 13:** **Device utilization summary of the reversible single precision floating point subtractor**

Device utilization summary (estimated values)			
Logic utilization	Used	Available	Utilization
Number of slice registers	97	19200	0%
Number of slice LUTs	698	19200	3%
Number of fully used LUT-FF pairs	97	698	13%
Number of bonded IOBs	100	360	27%
Number of BUFG/BUFGCTRLs	3	32	9%

### Power consumption of the proposed work

**Figure 28:**
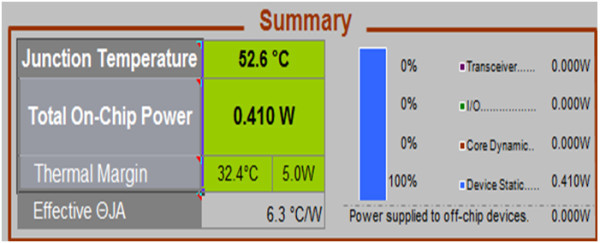
**Power consumption of the reversible 32-bit floating point subtractor.**

The power consumed by the proposed design is then estimated using Xilinx power estimator tool. Figure 28 shows the total on-chip power consumed by the system.

The total on-chip power consumed by the proposed 32-bit reversible floating point subtractor is 0.410 W.

## Conclusion

In this paper, an efficient reversible single precision floating point subtractor is designed with lesser number of garbage outputs, constant inputs and minimum number of transistors and has a latency of 2 clock cycles. An 8-bit and 24-bit reversible comparator is designed using the optimized 1-bit comparator with the reversible gates Reversible Gate1 and Reversible Gate2. An eight-bit and 24-bit reversible subtractor is designed using TR gates with less critical path delay. A 24-bit and a 25-bit reversible shift register is designed using the existing D-flip-flop and a 24-bit Reversible Leading Zero detector is designed using our proposed gate RG1. In effect, an efficient reversible single precision floating point subtractor is designed which will be very useful for the future computing techniques like ultra low power digital circuits and quantum computers. It is shown that the proposal is highly optimized in terms of number of reversible logic gates, number of garbage outputs, number of constant inputs and quantum cost. The future work is to use the proposed work in the design of reversible single precision floating point divider.
